# Melodic Contour Training and Its Effect on Speech in Noise, Consonant Discrimination, and Prosody Perception for Cochlear Implant Recipients

**DOI:** 10.1155/2015/352869

**Published:** 2015-09-30

**Authors:** Chi Yhun Lo, Catherine M. McMahon, Valerie Looi, William F. Thompson

**Affiliations:** ^1^Department of Linguistics, Macquarie University, Sydney, NSW 2109, Australia; ^2^HEARing Cooperative Research Centre, Melbourne, VIC 3053, Australia; ^3^ARC Centre of Excellence in Cognition and Its Disorders, Macquarie University, Sydney, NSW 2109, Australia; ^4^SCIC Cochlear Implant Program-An RIDBC Service, Sydney, NSW 2109, Australia; ^5^Department of Psychology, Macquarie University, Sydney, NSW 2109, Australia

## Abstract

Cochlear implant (CI) recipients generally have good perception of speech in quiet environments but difficulty perceiving speech in noisy conditions, reduced sensitivity to speech prosody, and difficulty appreciating music. Auditory training has been proposed as a method of improving speech perception for CI recipients, and recent efforts have focussed on the potential benefits of music-based training. This study evaluated two melodic contour training programs and their relative efficacy as measured on a number of speech perception tasks. These melodic contours were simple 5-note sequences formed into 9 contour patterns, such as “rising” or “rising-falling.” One training program controlled difficulty by manipulating interval sizes, the other by note durations. Sixteen adult CI recipients (aged 26–86 years) and twelve normal hearing (NH) adult listeners (aged 21–42 years) were tested on a speech perception battery at baseline and then after 6 weeks of melodic contour training. Results indicated that there were some benefits for speech perception tasks for CI recipients after melodic contour training. Specifically, consonant perception in quiet and question/statement prosody was improved. In comparison, NH listeners performed at ceiling for these tasks. There was no significant difference between the posttraining results for either training program, suggesting that both conferred benefits for training CI recipients to better perceive speech.

## 1. Introduction

Cochlear implants (CIs) are surgically implanted hearing devices that enable the perception of sound for most persons diagnosed with severe to profound deafness. Designed primarily for the purpose of speech perception, they are generally effective in quiet environments but less effective for perceiving speech in noisy environments [1] or for perceiving prosody [2, 3]. Prosody provides information such as the emotional state of a speaker and is used to transform linguistic content from statements to questions with the use of intonation. These difficulties are, in part, due to the lack of fine-structure processing in CI processing strategies that utilise temporal envelope, such as the Advance Combination Encoder (ACE) [4, 5]. As oral communication often occurs in the presence of complex and noisy acoustic environments, and prosodic components of speech convey important aspects of expression, these limitations can have a direct impact on social interactions and quality of life outcomes [6].

Advances in our understanding of neuroplasticity and learning capacity have led to interest in formal auditory training, with investigators proposing that it may form a component of comprehensive (re)habilitation [6, 7]. As some studies have demonstrated that normal hearing (NH) musicians are particularly adept listeners under challenging listening conditions such as noise [8, 9], the incorporation of music as a tool for improving language based tasks is a focus for many studies [9–11].

Using auditory brainstem responses (ABR), Parbery-Clark et al. [12] showed that NH musicians exhibited enhanced neural responses at the phoneme level for stop consonants /ba, da, and ga/, relative to nonmusicians. In addition, Strait and Kraus [13] also found that musicians were both faster and more precise than nonmusicians with encoding voice onset time (VOT) and second formant (F2) transitions, both of which contribute to the perception of stop consonants.

A study by Galvin III et al. [14] trained 11 CI recipients with an adaptive, PC-based melodic contour program for 30 minutes a day, with a time course varying between 1 week and 2 months for each participant. Posttraining results using a Melodic Contour Identification (MCI) task indicated that CI recipients' performance was improved between 15.5 and 45.4 percentage points. Recent research extending from this paradigm has investigated the use of melodic contours as training stimuli for CI recipients to improve speech perception tasks. In a preliminary study described in Patel [11], CI recipients were trained to play melodic contours on a piano keyboard 30 minutes a day, 5 days a week, for 1 month. The training stimuli consisted of 9 patterns of 5-note sequences that varied in the size of the intervals between consecutive tones (as used in [14]). Participants trained with intervals between 1 and 3 semitones, with the hypothesis that practise in this task should develop greater precision for MCI. While preliminary results (from two CI recipients) suggest that melodic contour training may improve intonation prosody and speech in noise perception, more evidence is needed to substantiate this finding. Thus, the present study is motivated by a need to provide additional evidence with a larger sample size and explore the transfer effects that melodic contour training may provide for enhancing the speech perception of CI recipients.

The OPERA hypothesis provides a theoretical framework that suggests why music training may drive perceptual speech gains [15]. These are as follows: overlap: acoustic features relevant to both speech and music are encoded on overlapping brain networks; precision: the requirements for music perception are higher than those for speech; emotion: the musical activity should elicit a strong positive emotion; repetition: the promo promotion of plasticity from repeated engagement of the neural network; attention: the focussed attention toward the task. When these conditions are met, there should be a flow on effect resulting in performance gains for speech perception.

Pitch-based tasks are a focus for many studies measuring music perception [16, 17], and music training programs have been used to improve pitch perception for CI recipients [18]. However, sounds are dynamic and multidimensional by nature, and different forms of music training may affect speech and music perception differentially. Therefore, a wider range of potential benefits, such as speed of processing, should be considered. An analysis of 5000 MIDI melodies suggests that mean note durations are approximately 280 ms [19], and an analysis of 16,000 syllables in American English suggests that mean syllable utterances are approximately 191 ms in length [20]. Thus the time available to extract cues is generally much shorter in speech than in music. Such a comparison can only be evaluated broadly, as there are many redundant cues that make speech accessible. The perception of various consonants that use VOT contrasts (e.g., the distinction between voiced and unvoiced stops /b/ and /p/) or formant trajectory discrimination (e.g., to identify stops within the voiced class such as /b/ from /g/) also relies on the extraction of cues across very short periods, between 5 and 50 ms, for effective perception [21]. As such, the exploration of shorter (and thus more difficult) note durations may be a mechanism for effective training. An emphasis on speed of processing is a differentiating factor for the present study and allows for an exploration of transferred skills, beyond the typical approach of manipulating pitch to adjust difficulty.

The purpose of the present study was to develop and evaluate two take-home, PC-based melodic contour training programs for CI recipients. The programs were adaptive and differentiated by two types of changes introduced in the stimuli: Interval: the interval size was adjusted and note duration was fixed; and Duration: the note durations were adjusted and interval size was fixed. As Patel's [11] results cannot disentangle effects related to the motor requirement of the piano playing task, we designed a purely perceptual training protocol. A key goal was to explore the transfer of nonlinguistic musical skills to specific aspects of speech perception. Using a baseline and posttraining paradigm, the relative efficacy of the two training programs was compared. It was hypothesised that both training programs should confer speech perception benefits to tasks that utilised pitch contours. Specifically, both programs would enhance speech in noise perception and prosodic cue perception due to improved F0 tracking, while consonant perception would be improved for participants assigned the duration program, due to greater speed of processing of VOT and F2 trajectories. The rationale was based on how short the transition period of VOT and F2 is (approximately 50 ms or less). Hence, identifying F2 is reliant on tracking the pitch over a short duration. As such, improvement in identifying melodic contours with shorter durations may have benefits for consonant perception, providing a specific advantage for consonant stops such as /b, d, g, p, t, k, m, and n/.

## 2. Materials and Methods

Approval for this study was granted by the Macquarie University Faculty of Human Sciences Human Research Ethics Subcommittee (reference: 5201400348).

### 2.1. Participants

Sixteen adult postlingually deafened CI recipients (11 female, 5 male) ranging in age from 26 to 86 (M = 58, SD = 15) and CI experience from 1 to 20 years (M = 9, SD = 7) participated in the study. All CI recipients used Cochlear Ltd. implants in unilateral, bilateral, or bimodal (with a hearing aid (HA)) configuration and were recruited from the Sydney Cochlear Implant Centre (SCIC). Eligibility required full time use of a CI, and at least 6-month CI experience. For performance reference purposes, 12 NH adults (6 female, 6 male) ranging in age from 21 to 42 (M = 27 years) were recruited from Macquarie University. All NH adults had hearing thresholds ≤30 dB hearing level (HL) measured in octave steps between 500 and 4,000 Hz, tested in a sound proof room. All participants were native Australian English speakers and did not have a significant impairment (such as a learning or cognitive disorder) that affected their ability for testing or training. Relevant demographic information can be found in Table 1.

### 2.2. Melodic Contour Training Program (MCTP)

**Figure 1:**
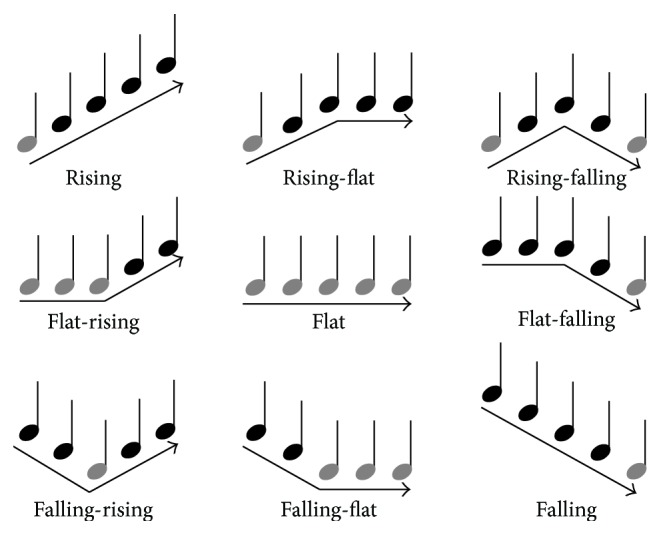
The 9 melodic contours used in the Melodic Contour Training Program. The lowest notes are marked in grey. From Galvin III et al. [14].

Two take-home PC-based training programs were created: MCTP (Interval) and MCTP (Duration). The training paradigm was adaptive with the stimuli becoming more difficult after a correct response and easier after every incorrect response. The program began at the easiest setting, and each change in difficulty was modulated by 1 level (one-up, one-down procedure), with a total of 7 levels of difficulty. The task was to identify a randomly selected melodic contour, at the designated difficulty level, using a four-alternative forced choice paradigm (4AFC). The melodic contours were sequences of 5 consecutive notes that formed a total of 9 patterns as used in Galvin III et al. [14].  Figure 1 shows the melodic contours used in the training programs.

The two programs differed by how difficulty was controlled. In the MCTP (Interval), note duration was fixed at 250 ms, and the interval size between consecutive notes was manipulated between 1 and 7 semitones that increased or decreased by 1 semitone. In the MCTP (Duration), interval size was fixed at 5 semitones, while the duration of each note was manipulated between 7 durations: 450, 350, 250, 200, 150, 100, and 50 ms. The lowest note in all stimuli was A4 for both programs; these are marked in light grey in Figure 1. The F0 range for the MCTP (Interval) was 440 to 2218 Hz, and the MCTP (Duration) was 440 to 1397 Hz. The stimuli were created using a Yamaha Disklavier Pro, providing a fairly realistic MIDI representation of an acoustic grand piano.

The program had two modes: “Practice” and “Training.” In Practice, participants were provided with all 9 melodic contours on their screen, and it was designed so that participants could practise listening to (and seeing) all 9 melodic contours available. The main task was the Training mode in which participants were presented with a melodic contour sound stimulus (which they could repeat), and four buttons representing answers, with one correct answer matching the presented contour, and three other options that were randomly selected from the pool of 9 contours. Feedback was provided after each response. If they were incorrect, the correct response would be highlighted, and they were then permitted (and encouraged) to listen for the differences between their selected and correct responses.

Data logging tracked the progress of each participant's session. For the MCTP (Interval), a melodic contour interval threshold was calculated, the interval size (measured in semitones) at which 50% of contours were correctly perceived. Similarly, for the MCTP (Duration), a melodic contour duration threshold was calculated. The thresholds for each session were averaged across each week of training.

### 2.3. Materials

The Australian Sentences Test in Noise (AuSTIN) is an adaptive speech in noise test developed specifically for Australian CI recipients [22]. Sixteen sentences were randomly selected and spoken by a female speaker in the presence of time-locked four-talker babble (4TB). In each session, two lists were completed, and a speech reception threshold (SRT, the signal to noise ratio at which 50% of words were correctly perceived) was calculated.

**Figure 2:**
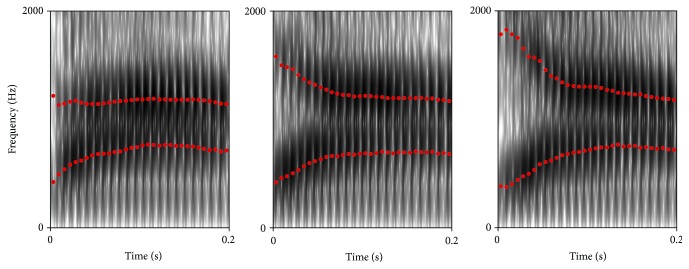
Spectrograms for voiced stop consonants /ba, da, and ga/ with F1 and F2 labelled.

A short Consonant Discrimination Test was developed for the purposes of this study, using a set of 12 commonly used consonants /pa, ta, ka, ba, da, ga, fa, va, sa, za, ma, and na/. The speech materials consisted of one male speaker and were validated for clarity and level-balance by two professional linguists. Lists consisting of 60 consonants in random order were created in two conditions: quiet and noise with 4TB (10 dB SNR). Spectrograms for voiced stop consonants are presented in Figure 2, highlighting F2 as the primary contrastive feature.

An individual subtest (turn-end reception) was selected from the Profiling Elements of Prosody in Speech-Communication (PEPS-C) [23], as a means to assess simple question and statement prosodic discrimination. Participants were presented with 16 single word utterances such as “carrot” or “milk” spoken by a female speaker that varied with intonation. Rising intonations indicated questions, while falling intonations indicated statements. With a 2AFC paradigm, participants were asked to indicate if the utterance was perceived as a question or a statement.

### 2.4. Procedures

Testing occurred in an acoustically treated test booth in the Macquarie University Speech and Hearing Clinic and in an acoustically treated room at SCIC, Gosford, NSW. The test battery was administered using a Toshiba Tecra R850 laptop. A Yamaha Audiogram 3 USB audio interface provided the sound signal and was connected to a Behringer Truth B3030A loudspeaker. Stimuli were presented at 65 dBA as measured with a sound level metre from the participant's listening position, located 1 metre in front of the loudspeaker. CI recipients were asked to use their regular, everyday settings and adjust their volume to a comfortable sound level on their Cochlear device and hearing aid. Once set, participants were requested to refrain from modifying any settings.

Following the baseline battery, participants were randomly assigned either the Interval or Duration program for the MCTP and provided instructions. There was an equal distribution of participants in each program. The training required the completion of one set of the “Training mode” (25 melodic contours, requiring approximately 15 to 30 minutes, depending on the participants' ability), 4 days a week, for a total duration of 6 weeks. All participants were provided with a set of Edifier M1250 USB powered loudspeakers to use during their training and instructed to train with their regular, everyday settings. Progress was monitored at 2 and 4 weeks, with contact through phone calls and email.

### 2.5. Statistical Methods

Analysis was performed with IBM SPSS Statistics version 21. Unless stated otherwise, each test was analysed using a repeated measures analysis of variance (ANOVA), with session (baseline and posttraining) as the within-subject factor and program (Interval or Duration) as the between-group factor. Additionally, the posttraining scores were compared between the CI group and the NH group using independent sample *t*-tests. All statistical tests used a criterion of 0.05 and all tests were 2-tailed.

## 3. Results

Group means and statistical data have been tabulated and are presented in Table 2.

While participants were randomly assigned a training program, to confirm there were no statistically significant differences in key variables between those assigned the Interval program compared with the Duration program, independent sample *t*-tests were calculated across age, CI experience, and all baseline scores. There were no statistically significant differences found; therefore the two groups were considered broadly equivalent prior to the training program.

Compliance was high, with data-logged results indicating that 13 participants completed the full 6 weeks of training. Additionally, there were no drop-outs. Two participants (1 and 7) were inconsistent, completing 4 weeks of the required training, but did compensate with extra sessions in those weeks trained. As such, their data was still included in the analyses. Unfortunately, the data-log recording training performance was corrupted and thus unavailable for participant 9. In summary, performance in the training programs was analysed for 15 participants (excluding participant 9), while baseline and posttraining speech perception measures included all 16 participants.

### 3.1. Melodic Contour Training Program (Interval)

**Figure 3:**
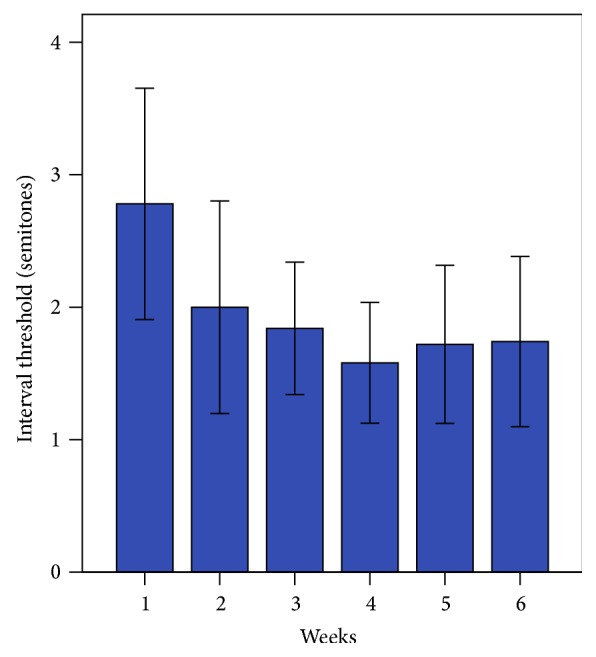
Week-to-week interval threshold scores for the Melodic Contour Training Program (Interval group). Error bars indicate 1 standard error.

Figure 3 shows the mean interval threshold (semitones) for each week of training. Using paired *t*-tests, the posttraining session threshold (measured at week 6, M = 1.7 ± 1.2 semitones) was significantly better compared with baseline (measured at week 1, M = 2.5 ± 1.7 semitones), *t*(6) = 2.75, *p* = 0.033, indicating that CI recipients were able to identify melodic contours with smaller interval sizes at posttraining than at baseline, with the greatest improvement found at week 4.

### 3.2. Melodic Contour Training Program (Duration)

**Figure 4:**
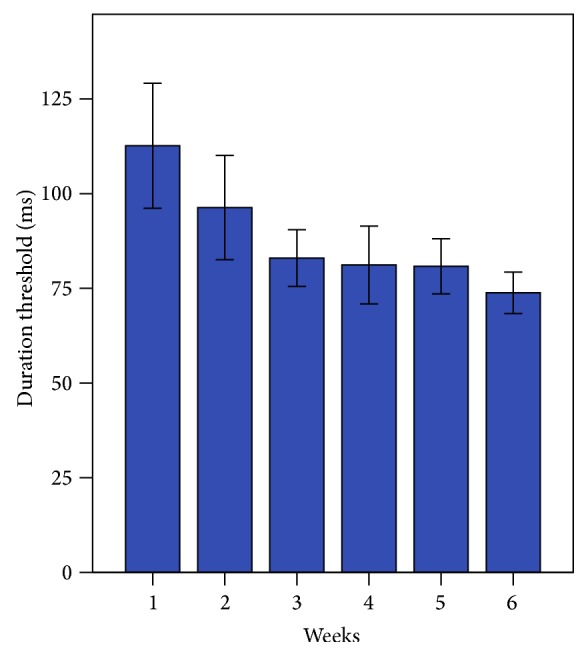
Week-to-week duration threshold scores for the Melodic Contour Training Program (Duration group). Error bars indicate 1 standard error.

Figure 4 shows the mean duration threshold (ms) for each week of training. Using paired *t*-tests, the posttraining session threshold (M = 79 ± 23 ms) was significantly better compared with baseline (M = 115 ± 39 ms), *t*(7) = 3.35, *p* = 0.012. These results indicate that CI recipients were able to identify melodic contours with shorter note durations at posttraining than at baseline. Ceiling performance was observed in 3 participants.

### 3.3. Australian Sentence Test in Noise (AuSTIN)

**Figure 5:**
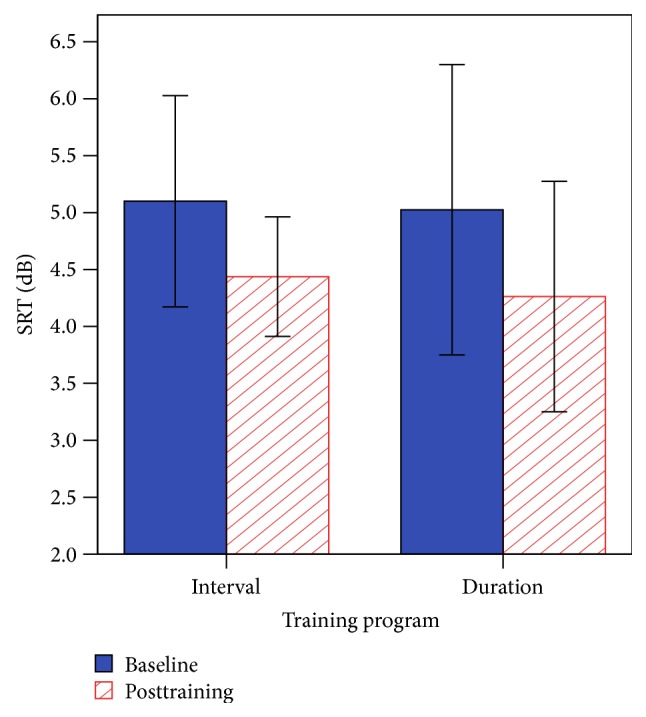
Baseline and posttraining SRTs for AuSTIN. Error bars indicate 1 standard error.

Figure 5 shows the mean SRTs for baseline and posttraining on speech in noise. The main effect of session was nonsignificant [*F*(1, 14) = 2.46, *p* = 0.139], the main effect of program was nonsignificant [*F*(1, 14) = 0.01, *p* = 0.925], and there were no interaction effects [*F*(1, 14) = 0.01, *p* = 0.914]. SRT scores at the posttraining session showed that the CI group was significantly higher (M = 4.4 ± 2.2 dB) compared with the NH group (M = −4 ± 0.9 dB), *t*(26) = 11.85, *p* < 0.001.

### 3.4. Consonant Discrimination in Quiet

**Figure 6:**
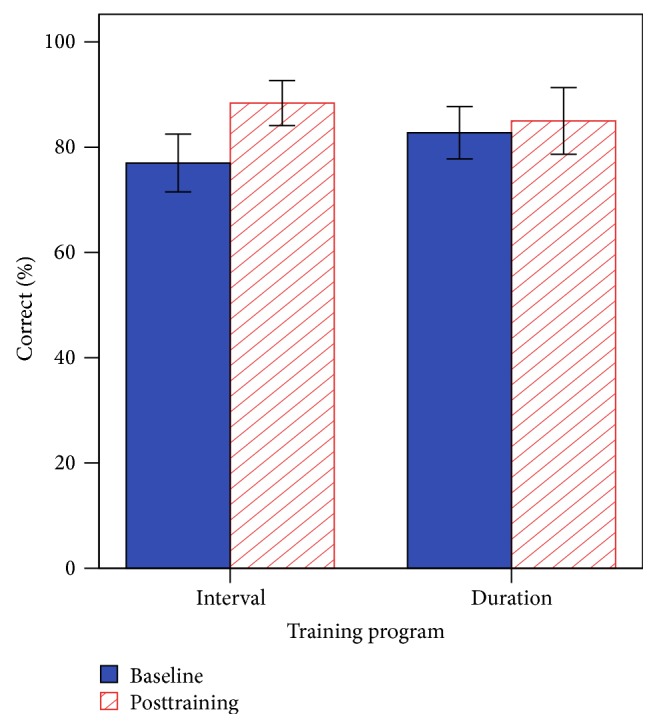
Baseline and posttraining performance for consonant discrimination in quiet. Error bars indicate 1 standard error.

The main effect of session was statistically significant [*F*(1, 14) = 6.00, *p* = 0.028], the main effect of program was nonsignificant [*F*(1, 14) = 0.03, *p* = 0.868], and there were no interaction effects [*F*(1, 14) = 2.69, *p* = 0.123]. Consonant scores in quiet at the posttraining session showed that the CI group was significantly lower (M = 87 ± 15%) compared with the NH group, with all NH individuals performing at ceiling (M = 100%), *t*(26) = −3.58, *p* = 0.003.  Figure 6 shows the mean scores (percent correct) for baseline and posttraining for consonant discrimination in quiet.

Further analysis using confusion matrices for individual consonants revealed that perceiving place of articulation was most improved for both training programs. To reconcile the analysis, only confusions greater than 10% (5 or more confusions) at baseline were considered.

**Figure 7:**
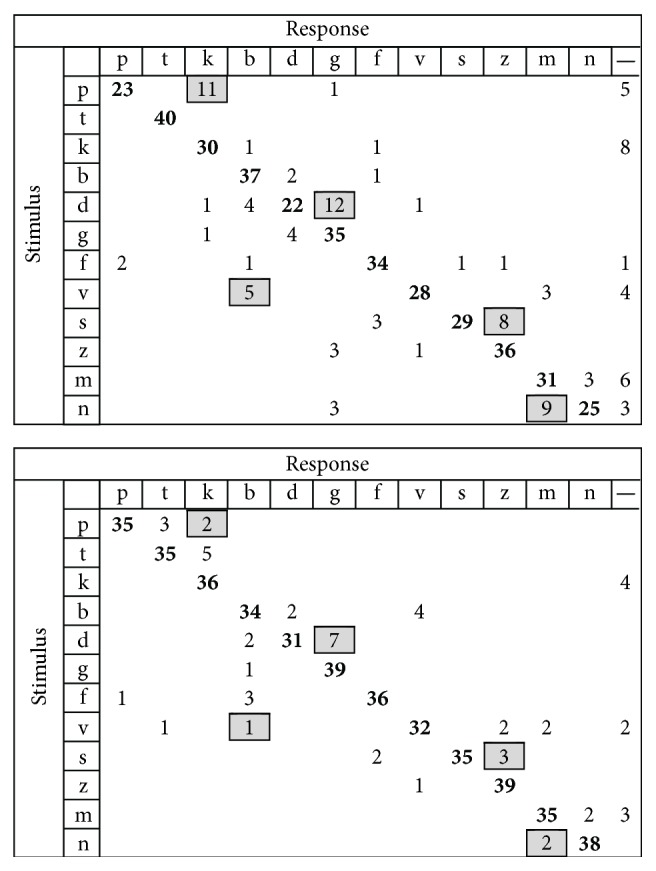
Confusion matrix for Interval group. Baseline is on top, posttraining at the bottom. Significant confusions in baseline have been marked in grey, and this is carried over to the posttraining matrix for easier visual identification of confusion decreases.

In the Interval group, analysis of individual consonants showed large improvements in the perception of stop consonants in which a 30% increase in accuracy was observed for /p/, a 23% increase for /d/, and an increase of 33% for /n/. A large reduction of confusions was observed for stop consonants, in which a 23% decrease was observed for /p/ perceived as /k/ and a 13% decrease was observed for /d/ perceived as /g/, in fricatives a 13% decrease was observed for /s/ perceived as /z/, and in the nasal stop an 18% decrease was observed for /m/ perceived as /n/. Pooled confusion matrices at baseline and posttraining for the Interval group are presented in Figure 7.

**Figure 8:**
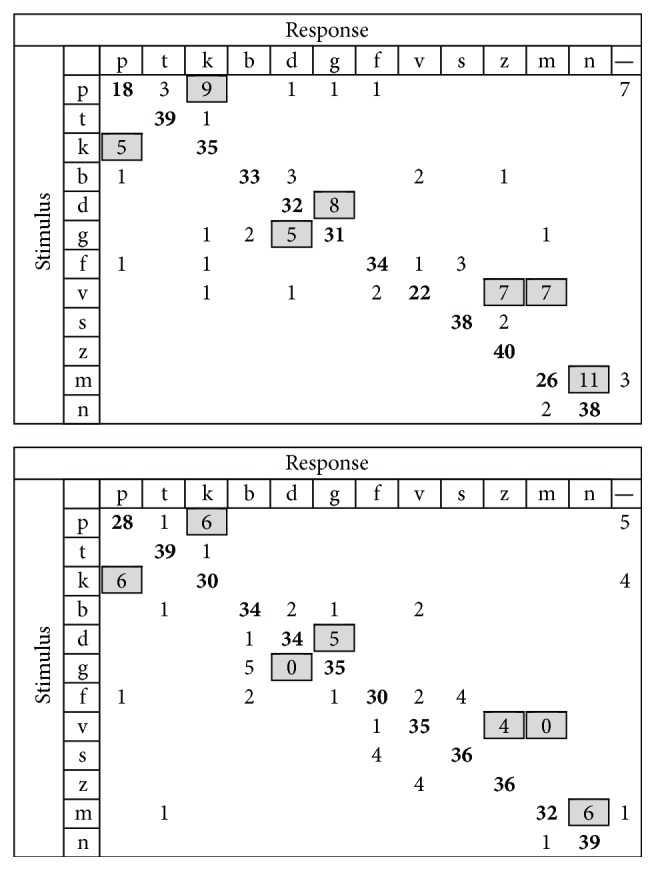
Confusion matrix for Duration group. Baseline is on top, posttraining at the bottom. Significant confusions in baseline have been marked in grey, and this is carried over to the posttraining matrix for easier visual identification of confusion decreases.

In the Duration group, analysis of individual consonants showed large improvements in the perception of stop consonants in which a 25% increase in accuracy was observed for /p/, a 33% increase was observed for /n/, and a 25% increase was observed for the fricative /v/. A large reduction of confusions was observed for stop consonants, in which a 13% decrease was observed for /g/ perceived as /d/, in fricatives an 18% decrease was observed for /v/ perceived as /m/, and in the nasal stop a 13% decrease was observed for /m/ perceived as /n/. Pooled confusion matrices at baseline and posttraining for the Duration group are presented in Figure 8.

### 3.5. Consonant Discrimination with 4TB

**Figure 9:**
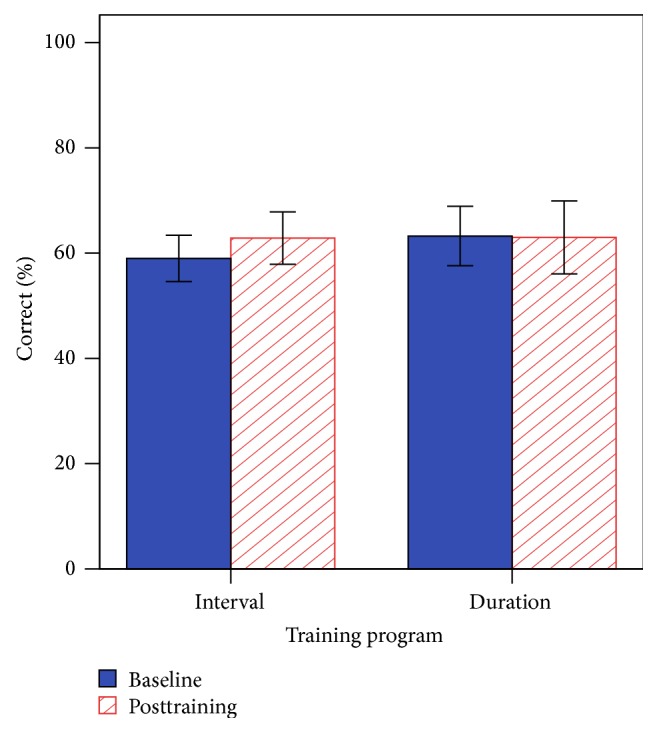
Baseline and posttraining performance for the consonant discrimination with 4TB. Error bars indicate 1 standard error.

The main effect of session was nonsignificant [*F*(1, 13) = 0.48, *p* = 0.500], the main effect of program was nonsignificant [*F*(1, 13) = 0.08, *p* = 0.779], and there were no interaction effects [*F*(1, 13) = 0.62, *p* = 0.444]. Consonant scores with 4TB at the posttraining session showed that the CI group was significantly lower (M = 63 ± 16%) compared with the NH group, with all NH individuals performing near ceiling performance (M = 99 ± 1%), *t*(26) = −9.08, *p* < 0.001. In the baseline session, participant 12 did not complete the task citing difficulty perceiving any consonants in noise. However, in the posttraining session after completion of training, the participant was able to complete the task, scoring 57% correct.  Figure 9 shows the mean scores (percent correct) for baseline and posttraining on consonant perception, with participant 12 included.

### 3.6. Profiling Elements of Prosody in Speech-Communication (Turn-End Reception)

**Figure 10:**
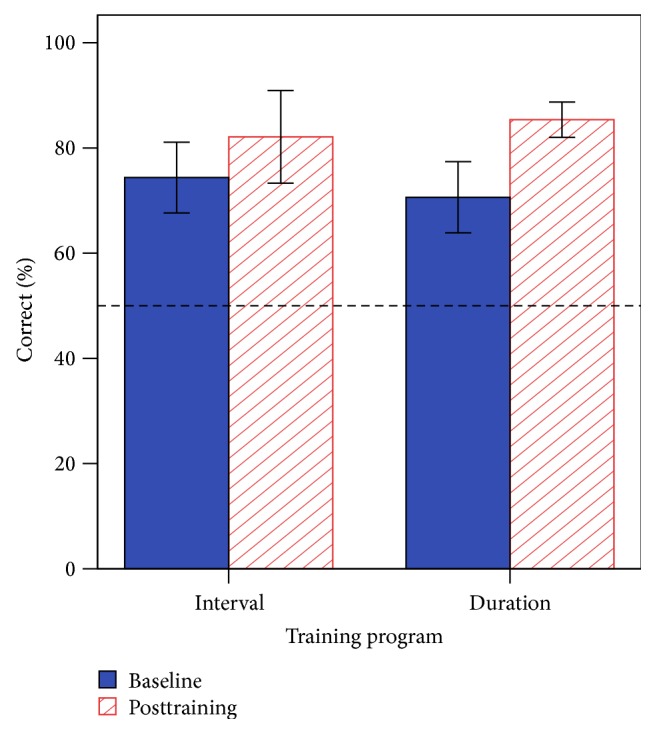
Baseline and posttraining performance for the PEPS-C (turn-end reception task). The dashed line indicates the chance score. Error bars indicate 1 standard error.

Figure 10 shows the mean percent correct for baseline and posttraining on question/statement prosody. The main effect of session was statistically significant [*F*(1, 14) = 9.31, *p* = 0.009], the main effect of program was nonsignificant [*F*(1, 14) = 0.01, *p* = 0.978], and there were no interaction effects [*F*(1, 14) = 0.90, *p* = 0.359]. Prosody scores at the posttraining session showed that the CI group was significantly lower (84 ± 18%) compared with the NH group, with all NH individuals performing at ceiling (100% accuracy), *t*(26) = −3.42, *p* = 0.004. These results indicate a significant posttraining improvement for prosody perception using intonation cues.

## 4. Discussion

The results indicate that melodic contour training can significantly improve some, but not all, aspects of speech perception in CI recipients. In particular, significant improvements for the perception of consonants in quiet and for the identification of questions and statements using only speech intonation cues were observed. Despite this, there were no significant group gains for speech in noise perception, or consonant perception in 4TB. Finally, and as expected, CI recipients performed more poorly than NH listeners in all tasks at pre- and posttraining measures.

Data-logged results from CI recipients indicate that MCI performance was significantly improved after six weeks of training in both Interval and Duration programs. However, for all tests, there was no significant effect for the type of program assigned to each participant. The greatest improvement was seen from week 1 to week 2 for both training programs, which may be an effect of familiarisation with the program. Maximum improvement with respect to interval and duration threshold was observed at weeks 4 and 6, respectively.

On all tests of speech perception there was no statistical difference between either of the training programs. These findings indicate that CI recipients were able to improve their pitch perception and temporal processing abilities in the context of MCI. While the relative efficacy between both mechanisms of interval size and note duration was nonsignificant, comparisons were difficult to make due to the small sample size, resulting in a lack of statistical power.

As the two training programs used significantly different musical mechanisms, it was surprising that the improvement in consonant perception in quiet had similar patterns for both training groups. In particular, confusions between place of articulation cues in voiced, unvoiced, and nasal stops were the most reduced, despite these cues being typified as the poorest speech production feature for CI recipients [24], which is an encouraging finding. As most improvement was found for stop consonant discrimination and as the F2 trajectory is the primary cue contrast, it is likely that recipients were better able to track F2 after training.

Both groups also showed significant improvement for the question-statement task that required cues of speech intonation. Firstly, it must be noted that the stimuli were single words consisting of one or two syllables, and the intonation pattern occurred over the final (or only) syllable. As such, there were no syntactic or semantic cues available and the improvement from training is most likely due to the mechanism of enhanced F0 tracking. However, it is possible that recipients also used duration and intensity cues across syllable boundaries as a distinction. Additionally, as question utterances rarely consist of just one word, the applicability of this enhancement to a more realistic question-statement identification task such as that with sentences, or in adverse conditions, is limited.

Based on preliminary results by Patel [11] that indicated the possibility of improvement for speech in noise perception as a result of melodic contour training, similar gains were anticipated for the current study. On the other hand, our findings indicate that, as a group, there was no significant improvement for consonant perception in noise, or with the perception of sentences in noise. Despite this, certain individuals showed large improvement in SRTs, although these were both bimodal listeners using a contralateral HA. This suggests that HA users, with more access to acoustic, F0, and fine-structure cues, may find melodic contour training particularly effective for speech in noise improvement. Aside from presenting data from a larger sample of participants, a key difference between Patel [11] and the present study was the removal of piano playing as the training paradigm. As such, the improvements found in the present study are inherently perceptual, as sensory-motor interactions (through the mapping of finger movements and musical notes) were not explicitly trained.

While our findings indicate some level of F0 improvement, primarily for intonation, such enhancement is only accessible in quiet, indicating that maskers significantly disrupt F0 cues for CI recipients that only have access to gross temporal envelope. Effective speech in noise perception is also reliant on auditory stream segregation processes to perceptually group and separate multiple sources [25]. As the melodic contours were a single-stream melody, it is unlikely that it would confer any benefit for segregation tasks.

The OPERA hypothesis suggests that music-driven speech gains are likely dependent on the type of training stimuli itself. Our results indicate that improvement to MCI, with an emphasis on pitch through the Interval program, and speed of processing with the Duration program both provide cues that transfer to more effective perception of stop consonants and speech intonation. As such, a training program manipulating both pitch and speed of processing difficulty may yield even greater improvement.

While there were overall group improvements for both training programs, there was considerable variation among individual participants, a common finding for CI studies. Ten of our participants were bilaterally implanted, and 3 participants were bimodal users. Two-ear listening allows for a binaural advantage, primarily improving spatially separated speech in noise tasks that require access to interaural cues to enhance localisation and segregation ability, relative to a unilateral CI [26]. On the other hand, as each of our speech perception tasks was delivered via one loudspeaker located at 0-degree azimuth, the main benefit of binaural devices was negated. Another benefit is binaural redundancy, whereby two ears (and binaural processing within the auditory system) integrate cues into a more salient speech signal, providing a small advantage of about 1 to 2 dB that may improve speech perception in adverse listening conditions [27]. It was not a main objective to evaluate differences between unilateral, bilateral, and bimodal configurations in this study, but these advantages should be noted. However, to maximise statistical power and generalisability, the inclusion criteria were extended to include all of these configurations, and we assumed that the difference between these groups would be nonsignificant for the measures evaluated, in a repeated measures design. Future studies could assess the effect of unilateral, bilateral, and bimodal devices on training efficacy.

The program had two tasks: Practice and Training, but data logging was only taken in the training mode. As such, the week-to-week improvements can only be interpreted broadly, as it is impossible to determine how much practice an individual completed. Additionally, participants were required to do at least 4 training sessions a week but were not discouraged from doing more. Nonetheless, irrespective of the rate of improvement, there were significant gains from baseline to posttraining.

This study was limited by a small sample, reducing the ability to evaluate subtle differences in the benefits of the two training protocols. Additionally, more robust baseline measures should be adopted ensuring stable asymptotic performance prior to training, such as introducing two or more spaced sessions prior to the training, as well as follow-up testing without training to ascertain if improvements are retained. Interpretation of cues is also made difficult without objective measures as complimentary evidence. The CI and NH groups were not age-matched, as the purpose was to provide a broad comparison across the speech tasks between the groups. However, it may be of interest to evaluate whether melodic contour training may improve older NH listeners' speech perception in noise. Certainly, cognitive abilities decline with age [28], and several studies show that music training is correlated with increased cognitive abilities [29]. Therefore it is possible that greater gains in speech perception might be found in older adults through improvements in cognitive ability.

## 5. Conclusion

In conclusion, the findings suggest that both musical mechanisms (intervals and durations) have had a beneficial outcome for CI recipients' perception of transition cues in quiet. These cues are most relevant for stop consonant distinctions and speech intonation, both of which derive the most advantage from melodic contour training. Masking effects, such as noise, significantly disrupt access to these cues, reducing the efficacy of melodic contour training in adverse listening situations.

## Figures and Tables

**Table 1:** Demographic information for Cochlear implant (CI) recipients.

ID	Age	Gender	CI/HA	Processor	Strategy	Number of electrodes activated	Unilateral/bilateral/bimodal	Number of years implanted	Training program
1	80	Female	L-CI24M R-CI24RE (CA)	L-CP810 R-CP810	ACE	L-16 R-22	Bilateral	20	Interval
2	26	Female	L-CI24RE (ST) R-HA	L-CP810	ACE	L-22	Bimodal	1	Interval
3	66	Male	L-CI422 R-HA	L-CP810	ACE	L-21	Bimodal	2	Duration
4	56	Female	L-CI24M R-CI24RE (CA)	L-CP810 R-CP810	ACE	L-18 R-22	Bilateral	14	Duration
5	35	Female	R-CI24RE (ST)	R-CP810	ACE	R-22	Unilateral	1	Duration
6	61	Male	L-CI24R (ST) R-CI24RE (ST)	L-CP810 R-CP810	ACE	L-22 R-14	Bilateral	12	Duration
7	47	Female	L-CI24RE (CA) R-CI24R (ST)	L-CP810 R-CP810	ACE	L-22 R-20	Bilateral	10	Interval
8	86	Female	L-CI24RE (CA) R-CI24RE (ST)	L-CP810 R-CP810	ACE	L-22 R-18	Bilateral	8	Interval
9	52	Female	L-CI24RE (CA) R-CI24RE (CA)	L-Freedom R-Freedom	ACE	L-22 R-18	Bilateral	10	Interval
10	54	Male	L-HA R-CI422	R-CP810	ACE	R-21	Bimodal	2	Duration
11	48	Male	R-CI512	R-CP810	ACE	R-22	Unilateral	4	Interval
12	69	Female	L-CI24RE R-CI24M	L-CP910 R-Freedom	ACE	L-21 R-22	Bilateral	15	Interval
13	66	Female	L-CI512 R-CI24RE (CA)	L-CP910 R-CP810	ACE	L-19 R-22	Bilateral	18	Duration
14	60	Male	L-CI24RE (CA) R-HA	L-CP810	ACE	L-22	Unilateral	2	Duration
15	67	Female	L-CI24RE (CA) R-CI24M	L-CP810 R-CP810	ACE	L-22 R-20	Bilateral	15	Interval
16	55	Female	L-CI422 R-CI22	L-CP900 R-Freedom	ACE	L-22 R-15	Bilateral	19	Duration

**Table 2:** Main effects of session, program, and interactions for all tests.

Test	*t* or *F* (df)	*p*
MCTP (Interval)		
Session	2.75 (6)	0.033^*∗*^
MCTP (Duration)		
Session	3.35 (7)	0.012^*∗*^
AuSTIN		
Session	2.46 (1, 14)	0.139
Program	0.01 (1, 14)	0.925
Session/program	0.01 (1, 14)	0.914
Consonant discrimination (quiet)		
Session	6.00 (1, 14)	0.028^*∗*^
Program	0.03 (1, 14)	0.868
Session/program	2.69 (1, 14)	0.123
Consonant discrimination (4TB)		
Session	0.48 (1, 14)	0.500
Program	0.08 (1, 14)	0.779
Session/program	0.62 (1, 14)	0.444
PEPS-C		
Session	9.31 (1, 14)	0.009^*∗*^
Program	0.01 (1, 14)	0.978
Session/program	0.90 (1, 14)	0.359

^*∗*^Indicates Significance at alpha = 0.05.
